# Differential modulation of human GABA_C_-ρ1 receptor by sulfur-containing compounds structurally related to taurine

**DOI:** 10.1186/s12868-018-0448-6

**Published:** 2018-08-03

**Authors:** Lenin David Ochoa-de la Paz, Martin González-Andrade, Herminia Pasantes-Morales, Rodrigo Franco, Rubén Zamora-Alvarado, Edgar Zenteno, Hugo Quiroz-Mercado, Roberto Gonzales-Salinas, Rosario Gulias-Cañizo

**Affiliations:** 1 Departamento de Bioquímica, Facultad de Medicina, Universidad Nacional Autónoma de México, Avenida Universidad 3000, Copilco Universidad, Cd. Universitaria, 04510 México City, México; 2 Departamento de Investigación APEC, Asociación para Evitar la Ceguera en México I.A.P. Hospital Dr. Luis Sánchez Bulnes, Fernández Leal 60, Col. La Concepción Coyoacán, 04020 Mexico City, Mexico; 3 División de Neurociencias, Instituto de Fisiología Celular, Universidad Nacional Autónoma de México, Circuito Exterior s/n, Coyoacán, Ciudad Universitaria, 04510 Mexico City, Mexico; 4 Redox Biology Center and School of Veterinary Medicine and Biomedical Sciences, University of Nebraska-Lincoln, Lincoln, NE 68583 USA

**Keywords:** Receptor modulation, GABA receptor, *Xenopus* oocytes, Sulfur-containing compounds, Taurine, Homotaurine, Hypotaurine, Isethionic acid

## Abstract

**Background:**

The amino acid taurine (2-Aminoethanesulfonic acid) modulates inhibitory neurotransmitter receptors. This study aimed to determine if the dual action of taurine on GABA_C_-ρ1R relates to its structure. To address this, we tested the ability of the structurally related compounds homotaurine, hypotaurine, and isethionic acid to modulate GABA_C_-ρ1R.

**Results:**

In *Xenopus laevis* oocytes, hypotaurine and homotaurine partially activate heterologously expressed GABA_C_-ρ1R, showing an increment in its deactivation time with no changes in channel permeability, whereas isethionic acid showed no effect. Competitive assays suggest that hypotaurine and homotaurine compete for the GABA-binding site. In addition, their effects were blocked by the ion-channel blockers picrotixin and Methyl(1,2,5,6-tetrahydropyridine-4-yl) phosphinic acid. In contrast to taurine, co-application of GABA with hypotaurine or homotaurine revealed that the dual effect is present separately for each compound: hypotaurine modulates positively the GABA current, while homotaurine shows a negative modulation, both in a dose-dependent manner. Interestingly, homotaurine diminished hypotaurine-induced currents. Thus, these results strongly suggest a competitive interaction between GABA and homotaurine or hypotaurine for the same binding site. “In silico” modeling confirms these observations, but it also shows a second binding site for homotaurine, which could explain the negative effect of this compound on the current generated by GABA or hypotaurine, during co-application protocols.

**Conclusions:**

The sulfur-containing compounds structurally related to taurine are partial agonists of GABA_C_-ρ1R that occupy the agonist binding site. The dual effect is unique to taurine, whereas in the case of hypotaurine and homotaurine it presents separately; hypotaurine increases and homotaurine decreases the GABA current.

**Electronic supplementary material:**

The online version of this article (10.1186/s12868-018-0448-6) contains supplementary material, which is available to authorized users.

## Background

Incorporation of sulfur into amino acids, proteins, enzymes, vitamins, and other biomolecules makes sulfur essential for biological systems [1]. In mammals, methionine is an essential amino acid, whereas cysteine, homocysteine, and taurine are semi-essential amino acids because they can be synthetized from methionine, sulfur, and serine via trans-sulfuration [1, 2].

Taurine (2-ethanosulfonic acid) is a ubiquitous, non-protein β-amino acid that abounds in different mammalian tissues. In the central nervous system (CNS) and retina, taurine is the second most abundant amino acid after glutamic acid, with a concentration in different species that ranges from 10 to 90 mM [3]. Taurine plays a role in different cell functions, as well as in neuronal migration and CNS development. In general, extensive experimental evidence reported relates taurine with cell volume and osmolality regulation [4–6]. In addition, taurine is involved in other physiological processes of the CNS and retina, such as modulation of Ca^2+^ channels and neurotransmission [7–11]. Other physiological functions of taurine not described in retina are antioxidant defense, phase II detoxification reactions and a role as a neurotrophic factor. GABA (γ-aminobutyric acid) plays a key role in neurotransmission. For example, in the retina, GABA modulates transmission of information flowing from photoreceptors to the brain. GABA performs its inhibitory action via two types of receptors: (1) ionotropic, designated GABA_A_ and GABA_C_ (also known as GABA_A_-ρ); and, (2) metabotropic, named GABA_B_, all of them with different molecular and pharmacological properties [12–15]. GABA_C_ receptor is particularly interesting because it is insensitive to barbiturates, benzodiazepines, bicuculline, and baclofen (modulators and inhibitors of GABA_A_ and GABA_B_ receptors, respectively) [16–20] but sensitive to picrotoxin and Methyl(1,2,5,6-tetrahydropyridine-4-yl) phosphinic acid (TPMPA) [21].

GABA_A_ receptors are heteropentamers, constituted by α, β, γ, and δ subunits; the combinations of these subunits determine the pharmacological and physiological properties of the receptor, and the α1/β2/γ2 is the most common combination found in the CNS and retina [22, 23]. GABA_C_ receptors are composed of the ρ1, ρ2, and ρ3 subunits, each of them capable of forming homomeric functional receptors when expressed heterologously. However, the native composition of these receptors in neurons is unknown. In the CNS, evidence of the expression of GABA_C_ receptors has been demonstrated in cerebellar Purkinje neurons and in the amygdala [24–26]. Experiments “in vivo” using specific antagonists of GABA_C_ receptors directly applied into the amygdala, suggest that GABA_C_-mediated activity participates in the modulation of fear and anxiety [26]. Although the role of GABA_C_ receptors in the retina is still poorly understood, its function is tightly modulated by intracellular cascades triggered by neuroactive molecules and their receptors, and they are thought to play a major role in visual signaling [19, 20, 27, 28]. While GABA_A_ receptors (α1/β2/γ2) are distributed in all neuronal types of the retinal circuit, GABA_C_ conformed by ρ1 and ρ2 subunits are located in bipolar neurons, where they downregulate NMDA receptors and, consequently, modulate transient glutamate release in response to light. In addition, amacrine and ganglion cells express GABA_C_ receptors [29–31].

Taurine exerts its inhibitory effect via activation of GABA_A_ and glycine receptors, but has less affinity compared to the specific agonists of each receptor [10, 11]. However, the mechanisms by which taurine regulates ionotropic GABA receptors remain to be determined. A negative and positive modulation of the GABA-induced current in oocytes heterologously expressing human GABA_C_-ρ1 receptor, exposed at lower and higher taurine concentrations was reported [32]. These results suggest that taurine acts in a dual way and may compete with GABA for the same binding site. Our study aimed to determine if the dual effect of taurine observed on GABA_C_-ρ1R was associated with the molecular structure of taurine. To confirm this hypothesis, we used homotaurine (Homo), hypotaurine (Hypo), and isethionic acid (IA), all sulfur-containing compounds structurally related to taurine (SCC-tau), but with some differences in their chemical structure. For instance, unlike taurine, Homo has one additional carbon and the sulfonic group (SO_3_H) is in *Cis* position with respect to the amino (NH_2_) group; Hypo has a sulfinic group (SO_2_H) in *Trans* position with respect to the NH_2_ group, and IA has a hydroxyl group instead of NH_2_. Some of these compounds have been widely used to better understand the physiological functions of taurine and, likewise, to evaluate compounds that may be therapeutically applied in some diseases such as diabetes, alcoholism, ischemia, and others [33–35]. Although a variety of taurine analogues have been developed, their effects are still unknown in terms of structure–function in various systems [36].

## Results

**Fig. 1:**
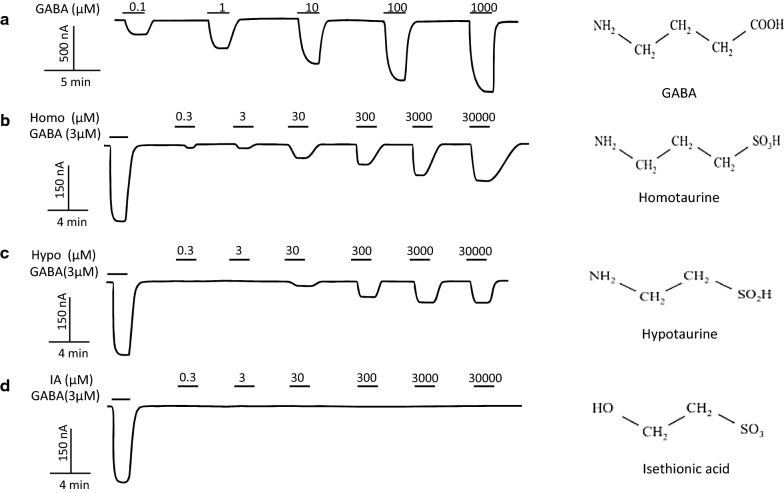
Activation by GABA and SCC-tau of GABA-ρ1R heterologously expressed in oocytes. **a** Control: oocyte exposed to GABA at several concentrations (3–1000 µM); **b**–**d** Oocytes perfused with SCC-tau at the concentrations indicated. In each experiment the oocyte was first exposed to a GABA concentration equal to GABA’s EC_50_. The horizontal bars indicate the period of time when the compound was applicated. Oocytes were voltage-clamped at − 60 mV and inward currents are denoted as downward deflections. Chemical structure of GABA (**a**) and SCC-tau (**b**–**d**), are represent at right of each representative trace

**Fig. 2:**
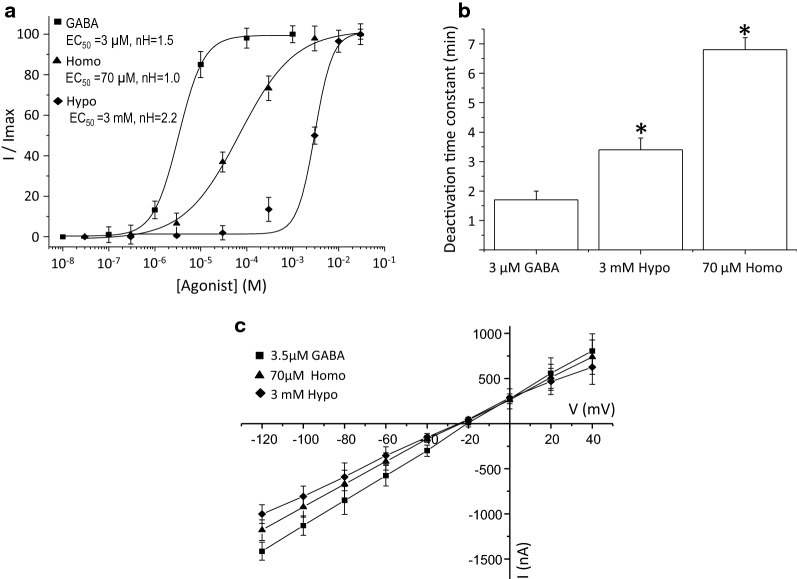
GABA- and SCC-tau-induced currents in oocytes heterologously expressing GABA_C_-ρ1R. **a** Dose-response relationship showing the EC_50_ and Hill coefficient for each agonist. Data were normalized to the maximal current (I) obtained for each agonist. **b** Deactivation constant (τ_deac_) of currents activated by GABA, Hypo, and Homo. The difference in the deactivation constants between, GABA-, Hypo- or Homo-induced currents, was significant when P < 0.05. **c** Current–voltage relationship of GABA-, Homo-, and Hypo-induce currents at the indicated concentrations. Dose-response relationship (**a**) was constructed by measuring the maximum response evoked by each agonist concentration (see methods), for **b** and **c**, data are given as the mean ± S.E. from at least 8 oocytes (n = 8) from 3 frogs (N = 3)

GABA_C_-ρ1 receptor (GABA_C_-ρ1R) heterologously expressed in oocytes generated typical non-desensitizing GABA currents (Fig. 1a) when SCC-tau was perfused onto the same oocyte; Homo- and Hypo- elicited a response in a concentration dependent manner. Both compounds gate the ion-channel at micromolar concentrations (Fig. 1b, c). In contrast, IA did not show any affect at the concentrations tested (Fig. 1d). Similar to the currents induced by GABA, Homo- and Hypo-induced well-maintained currents and did not desensitize, even after long exposure. Despite the lack of differences between the magnitude of the current elicited by Homo- and Hypo-, these SCC-tau show a clear difference in the EC_50_ of 70 ± 1.1 µM for Homo and 3 ± 1.5 mM for Hypo (Fig. 2a). The response induced by Homo and Hypo did not correlate with changes in the activation time of the currents (τ_act_). Nevertheless, Homo showed an increase in deactivation time (τ_deac_). The τ_act_ and τ_deac_ for GABA alone were 1 ± 0.2 min and 1.7 ± 0.5 min, respectively. In the presence of Hypo, τ_act_ was 1.5 ± 0.1 min and t_deact_ was 3.4 ± 1.1 min, whereas for Homo τ_act_ was 1.8 ± 0.2 min and τ_deac_ was 6.7 ± 2.2 min (Fig. 2b). However, we did not observe any permeability changes of the channel activated, as suggested by their inversion potential, which was − 21 ± 1.0 mV for 3.5 µM GABA and − 25 ± 2.1 mV and − 26 ± 1.0 mV for 70 µM Homo and 3 mM Hypo, respectively (Fig. 2c). In all cases, the current–voltage (I–V) relationship was linear within the range explored (− 120 to + 40 mV), indicating that activation of GABA_C_-ρ1R by SCC-tau was voltage-independent and did not change the ion selectivity of the channel.

**Fig. 3:**
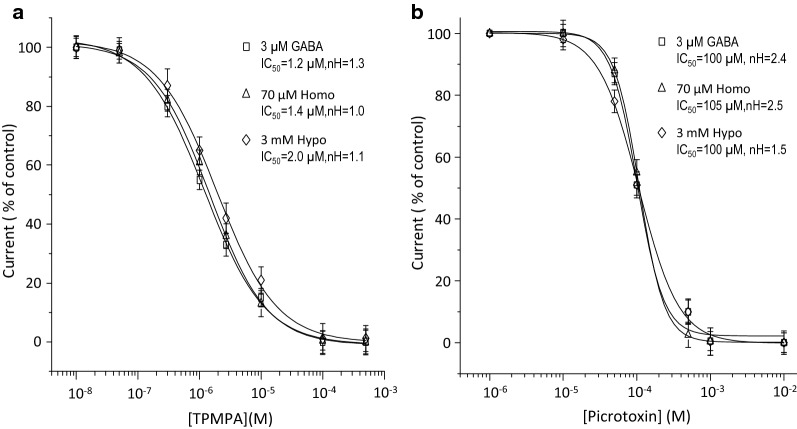
Pharmacological modulation of GABA- and SCC-tau-induced currents by TPMPA (**a**) and picrotoxin (**b**). Currents were normalized to the maximum amplitude elicited by GABA or SCC-tau in absence of inhibitors. Data points are the mean ± S.E. from at least 7 oocytes (n = 7) from 3 frogs (N = 3)

We also analyzed if the currents activated by Homo and Hypo were sensitive to TPMPA, a highly specific and selective antagonist of GABA_C_-ρ1R [21]. Figure 3 shows an IC_50_ of 1.2 ± 0.1 µM, 1.4 ± 0.1 µM or 2.0 ± 0.2 µM TPMPA when the receptor was activated by 3 µM GABA, 70 µM Homo or 3 mM Hypo, respectively (Fig. 3a). Hill coefficients were 1.3 ± 0.2 µM (GABA), 1.0 ± 0.1 µM (Homo), and 1.1 ± 0.8 µM (Hypo). It is well known that TPMPA competes with GABA for the same binding-site, so since the IC_50_ of TPMPA values are similar; it is probable that SCC-tau, Homo and Hypo, share the same binding site as GABA in the GABA_C_-ρ1R. As expected, picrotoxin, an allosteric antagonist of ionotropic GABA receptors [37, 38], irreversibly inhibits the currents activated by 3 µM GABA, 70 µM Homo or 3 mM Hypo in a dose-dependent manner, with an IC_50_ of 100 ± 0.1 µM, 105 ± 0.3 µM, and 100 ± 0.8 µM, respectively (Fig. 3b).

**Fig. 4:**
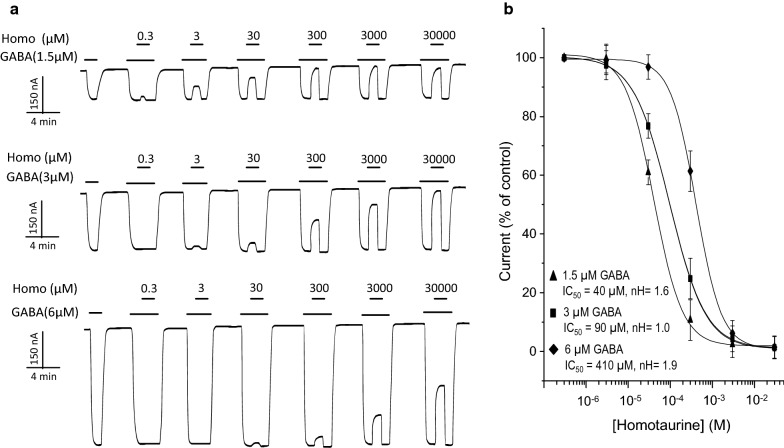
Effect of Homo on GABA-induced currents in oocytes heterologously expressing GABA_C_-ρ1R. **a** Representative traces of currents induced by 1.5, 3, and 6 µM GABA and co-applied with Homo at the indicated concentrations. **b** Homo dose-response relation of currents elicited by 1.5, 3, and 6 µM GABA. The currents were normalized to the maximum amplitude elicited by the agonist in absence of modulators. Data points are the mean ± S.E. from at least 9 oocytes (n = 9) from 4 frogs (N = 4)

Figure 4 shows that during GABA-induced activation of the receptor, Homo reduced the currents when co-applied at different concentrations (3 µM to 30 mM). The effect of Homo on the GABA-induced current changes depended on GABA concentrations. Figure 4a shows that at a concentration of 1.5 µM and 3 µM of GABA, Homo decreased GABA-activated currents. However, at a higher concentration of GABA (6 µM), the effect of Homo diminished; the IC_50_ for Homo were 40 ± 1.0 µM, 90 ± 1.5 µM, and 410 ± 2.9 µM at 1.5 µM, 3 µM and 6 µM GABA, respectively (Fig. 4b). In all cases, the GABA-induced currents without SCC-tau, increased in a dose-concentration fashion.

**Fig. 5:**
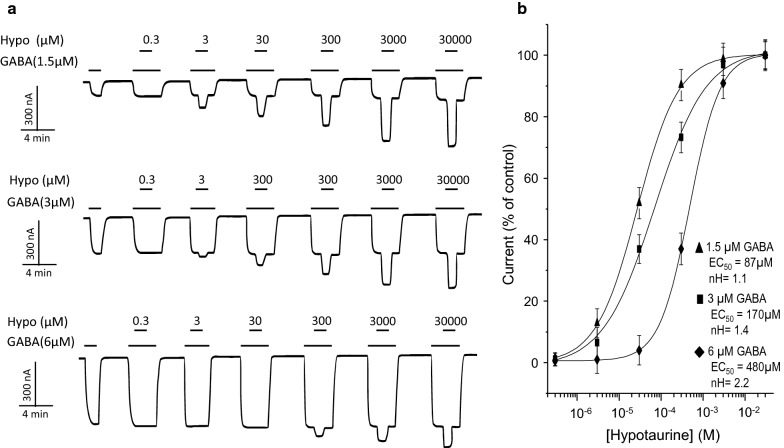
Effect of Hypo on GABA-induced currents in oocytes heterologously expressing GABA_C_-ρ1R. **a** Representative traces of currents induced by 1.5, 3, and 6 µM GABA and co-applied with Homo at the indicated concentrations. **b** Hypo dose-response relation of currents elicited by 1.5, 3, and 6 µM GABA. The currents were normalized to the maximum amplitude elicited by the agonist in absence of a modulator. Data points are the mean ± S.E. from at least 9 oocytes (n = 9) from 4 frogs (N = 4)

We also analyzed whether the stimulatory effect of Hypo changed by the extracellular GABA concentration and we found that unlike Homo, Hypo potentiated GABA currents. As observed in Fig. 5a, at 1.5 µM GABA, Hypo induced a current increase, that was even greater than the current activated by GABA alone. At 3 µM GABA, the effect of Hypo was still present almost with the same characteristics as 1.5 µM GABA; however, at 6 µM GABA, the increase induced by Hypo on GABA currents diminished. GABA-induced current increased in a dose-concentration fashion. The EC_50_ values obtained for Hypo were 87 ± 1.5 µM, 170 ± 1.7 µM, and 480 ± 2.1 µM for 1.5 µM, 3 µM, and 6 µM GABA, respectively (Fig. 5b). These results suggest a competitive action of SCC-tau with GABA for the same binding site (Additional file 1: Figure S1). In the case of IA, we did not observe any effect (data not shown).

### Molecular modeling

**Fig. 6:**
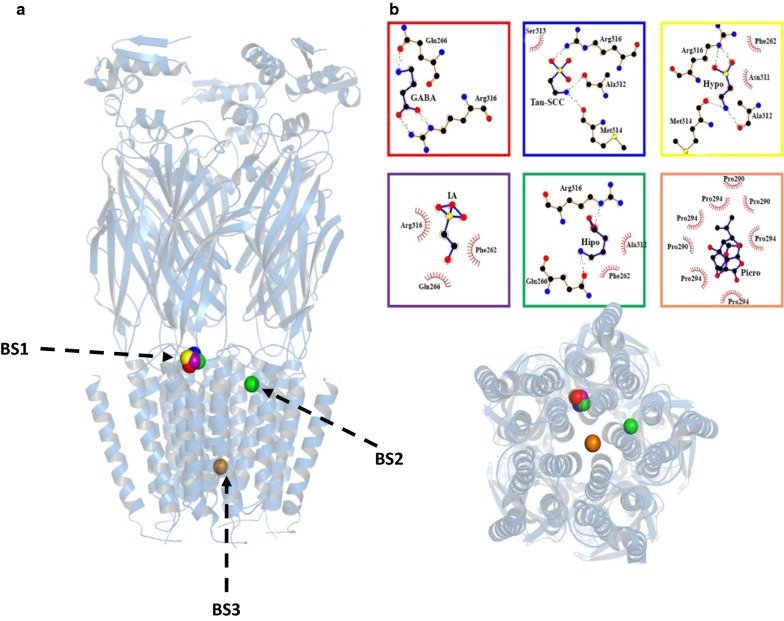
Structural models of GABA_A_ side view (**a**) and top view (**b**) of the receptor and results of docking with different ligands, represented by colored spheres GABA (red circle), Tau-SCC (black circle), Hypo (orange circle), IA (violet circle), Homo (green circle), and Picro (brown circle). The structures were drawn using the PyMOL and LIGPLOT v.4.5.3 programs

To prove theoretical studies on the interactions of the different ligands with the human GABA_C_-ρ1receptor, we performed homology modeling. We selected as target the structure of the GABA_C_-ρ1 R-beta3 homopentamer, since it has coverage of around 70% and an identity of 48%. The original sequence of this gene (GenBank: AAA52509.1) has an initial segment of 44 amino acids with a disordered/unstructured identity. We constructed this segment using an “ab inition” modeling, which shows good structural correlation. With this segment and the crystallographic structure, the GABA_A_-ρ1R model was constructed and validated (Additional file 2: Figure S2). This model presents an initial segment corresponding to the transmembrane section, which may have an effective recognition function. Subsequently, using this constructed model, we performed docking studies with different ligands (GABA, Homo, Hypo), IA, and one GABA_A_ receptor antagonist (picrotoxin). For docking probes, we included taurine to compare the binding sites with SCC-tau and GABA. Figure 6 shows the results obtained after performing 1000 runs for each compound, observing three different binding sites. In the first one we located (BS1) GABA, taurine, Homo, Hypo, and IA; the second binding site (BS2) apparently is exclusive for Homo, additional to the site it shares with GABA and SCC-tau, and the third one (BS3) corresponds to picrotoxin, located inside the receptor. The binding parameters (K_i_) obtained by AutoDock4 are in the mM range, except for picrotoxin that is of a lesser order of magnitude. The order of affinity predicted is as follows: picrotoxin (0.012 mM) < IA (3.25 mM) < Homo (3.56 mM) < GABA (8.48 mM) < Hypo (10.50 mM) < taurine (10.86 mM). There are different binding sites at the GABA_C_-ρ1R that function as modulators of the activity of this receptor. Therefore, we complemented the experimental results with theoretical studies, observing some correlation, such as: (1) the structural relationship between Homo and Hypo to activate GABA_C_-ρ1R, demonstrates that Homo is a more potent agonist than Hypo; also, it has a lower K_i_ than Hypo; (2) competition experiments between TPMPA and GABA by GABA_C_-ρ1R indicate that Homo and Hypo (even taurine), interact at the same site (BS1), supporting the data obtained by docking; (3) Homo has a second binding site (BS2), that is not used by GABA or SCC-tau; and (4) picrotoxin, an allosteric antagonist of ionotropic GABA receptors, its binding was predicted in the anion channel of the receptor (BS3), a unique site not used by the SCC-tau compounds with a strong affinity according to literature reports for this blocker [37, 38].

## Discussion

The use of SCC-tau, that possesses only some of the properties of taurine, has increased our understanding about the role of this β-amino acid in the cell physiology. Previous reports show that SCC-tau modulates the inhibitory action triggered by GABA [39–41]. However, the structural characteristics that determine these effects are unclear. In this work, we report that even though Homo, Hypo, and IA share similar core structures, Homo was a potent partial agonist of GABA_C_-ρ1R compared to Hypo. This observation could indicate that the presence of an additional carbon and the SO_3_H group in *Cis* position with respect to the NH_2_ group in Homo´s structure allows it to form a moiety that enhances the affinity and binding of Homo for GABA_C_-ρ1R. In contrast, it is probable that the absence of an NH_2_ group and the short carbon skeleton are responsible for the non-response effect with IA.

Oocytes that heterologously express the GABA_C_-ρ1R can generate currents when exposed to GABA, Homo, or Hypo. When SCC-tau and GABA were co-applied, we observed a concentration-dependent decrease (Homo) or increase (Hypo) of GABA induced-currents. Interestingly, in both cases, we did not observe a *dual* action of Homo and/or Hypo in the modulation of GABA-induced currents, as in the case of taurine [32]. In this sense, unlike the human glycine receptor (Gly-α1R), where taurine and other β-amino acids act like agonists or antagonists [42], in GABA_A_-ρ1R only taurine follows this pattern. One interpretation for this lack of *dual* effect of Homo and Hypo is that probably its action it is not associated to a change in the molecular structure of the compounds [42], and the positive and negative modulation on the GABA-induced current could be a result of the occupation of the agonist binding sites in the binding pocket by each of these compounds. A second possibility is and effect of either negative or positive cooperativity between GABA-Homo or GABA-Hypo.

In the case of taurine, it was previously reported that it generates a *dual* effect on GABA-induced currents, suggesting that this β-amino acid (at low concentrations) can compete with GABA for the same binding site [32]. This hypothesis is reinforced with our molecular model, where Hypo, Homo, IA, taurine and GABA share the same binding site. The fact that taurine inhibits GABA response at high concentrations [32], just like Homo in this work, could be due to an interaction of taurine and Homo with a second binding site.

These data suggest a competitive behavior of SCC-tau and, thus, reflects an antagonistic binding within a common ligand-binding pocket. The Hill coefficient obtained in both the negative and positive modulations generated by Homo and Hypo, respectively, indicates a contribution of GABA displacement despite its affinity to the binding site. This structural phenomenon would allow the interaction of SCC-tau in different binding conformations, inducing agonist or antagonist effects.

GABA induced-currents were modulated when SCC-tau and GABA were co-applied, while Homo reduced, Hypo increased the currents. This effect is explained with the data obtained from the docking, where Homo presents a second site with a greater binding probability, different from the binding site it shares with GABA; these two sites are located at different places of the receptor. Therefore, considering that GABA shares the same binding site as Hypo and Homo, this would confirm the fact that they compete for the same binding site as reflected in the experimental protocol. However, in the case of Homo, it does not share the second binding site with GABA, so there is no competition between them. Therefore, it is likely that this second Homo binding site may act as a negative regulatory site of the GABA_C_-ρ1R, when GABA activates the receptor.

Homo acts like an antagonist of GABA- and Hypo-induced currents; however, is a partial agonist at high concentrations without GABA or Hypo. This could be explained by negative cooperativity between Homo and GABA or Hypo, and this interaction is possibly due to a dependence of the second Homo binding site on the action of the agonist, that is available only when the first site (BS1) is previously occupied by GABA or Hypo. Therefore, it is likely that the affinity of Homo depends on the previous binding of a first agonist, which changes the conformational structure of the receptor.

## Conclusions

Interest in receptors for γ-aminobutyric acid, the major inhibitory transmitter in the central nervous system, has been development over the last four decades. Given their widespread distribution, lower abundance and relative simplicity compared to GABA_A_ and GABA_B_ receptors, GABA_C_ receptors are attractive drug targets. GABA_C_ receptors pharmacology is different from GABA_A_ and GABA_B_ receptors, therefore, the development or characterization of novel compounds for GABA_C_ receptor is imperative. This will allow to determine with certainty the function of this receptor in the central nervous system. In this sense, small molecules such as SCC-tau are compounds with great potential for this purpose. Here, we observed that SCC-tau are partial agonists of the GABA_C_-ρ1R that occupy the agonist binding site. The dual effect observed with SCC-tau was present in a separate way: while Hypo increases, Homo decreases the GABA-currents. These observations suggest that taurine could induce the dualistic effect by a change in the molecular structure in response to taurine concentration. However, the fact that Hypo and Homo act differently on GABA-currents could potentially be used to design pharmacological tools for the modulation of GABAergic receptors.

## Methods

### Expression of homomeric human GABA_C_-ρ1 receptors in Xenopus laevis oocytes

All the animals were handled in accordance with guidelines of the National Institutes of Health Guide for Care and Use of Laboratory Animals and with the approval of the Institutional Animal Care and Use Committee of the National University of Mexico. We used Ethyl 3-aminobenzoate methanesulfonate (MS-222) at 0.17% for 30 min, to anesthetize the *Xenopus laevis* frogs. The oocytes were manually removed and enzymatically defolliculated at room temperature using collagenase type I (0.3 μg/μl) for 45 min. Later, oocytes were kept in Barth’s medium (in mM): 88, NaCl; 1, KCl; 0.33, Ca_2_ (NO)_3_; 0.41, CaCl_2_; 0.82, MgSO_4_; 2.4, NaHCO_3_; 5, HEPES; pH 7.4 and 0.1 mg/ml gentamicin sulfate. After 24 h, 0.5 μg/μl of human GABA_C_-ρ1R mRNA was injected in vegetal hemisphere of the oocyte, and electrophysiological recordings were obtained 2–3 days after injection.

### Voltage-clamp recordings

Membrane currents elicited by GABA and SCC-tau were recorded using the two-microelectrode voltage-clamp technique. Oocytes were placed in a chamber, with a volume of 500 μl, and impaled with two microelectrodes previously filled with 3 M KCl (0.5–2.5 MΩ) and clamped at − 60 mV. To determine the equilibrium membrane potential of the agonist action, I–V relationships were constructed by stepping the oocyte’s membrane potential from − 60 to − 120 mV for 1 s and then from − 60 to + 40 mV (in 20 mV steps) in the absence or presence of GABA or SCC-tau (Homo, Hypo, IA). All recordings were done at 20–23 °C in a chamber continuously perfused at 5–10 ml/min of Ringer solution (in mM): 115, NaCl; 2, KCl; 1.8, CaCl_2_; 5, HEPES, pH 7.4. All drugs were purchased from SIGMA-ALDRICH (San Louis Missouri, USA). The stock solution of GABA (1 M), Homo (0.5 mM), Hypo (0.5 mM) or IA (0.5 mM) was kept frozen until thawed for the experiments. The pH of all solutions was adjusted to 7.4.

### Data analysis

Results are reported as mean ± S.E. of the values obtained from several cells, each value being the average of measurements in different cells. Data from each experiment were collected from at least seven oocytes. Agonist concentration–response curves were constructed by measuring the maximum response evoked by each agonist concentration. The half-maximal concentration (EC_50_) and Hill coefficient (n_H_) of GABA, Hypo, and Homo were estimated for each curve by fitting the data to the logistic type equation (Origin 6.0, Northampton, MA): ${\text{A}} = {\text{A}}_{ \text{max} }/(1 + 10^{{{{ [ {\text{logEC}}_{50}}}^{{{-}[{\text{agonist] nH}}}}}})$. The half-inhibitory concentration (IC_50_) of Homo was estimated by fitting the following equation: ${\text{A}} = {\text{ A}}_{ \text{max} } /(1 + 10^{{[[{\text{agonist}}] - {\text{logEC}}_{ 5 0} }} )$. Differences among groups were statistically analyzed by ANOVA and Tukey–Kramer post-test, and were considered significant when P < 0.05. Control responses to GABA were obtained before and after each drug application to account for possible shifts in the amplitude of the control current. To determine the time constants for the activation (τ_act_) and deactivation (τ_deac_) of GABA-, Homo- and Hypo-currents responses, a decay function of the form: *I*(τ) = exp(− *t*/τ _d_) + C, where *I* is the current and *t* is time, was fit to the experimental data (Origin 6.0 software; Northampton, MA). Differences between groups were statistically analyzed by ANOVA and a Tukey–Kramer post-test, and were considered significant at the level P < 0.05.

### Homology modeling of Type A gamma-aminobutyric acid (GABA_A_) receptors (Homo sapiens)

This homology modeling started with the retrieval of the amino acid sequence of GABA_A_ (GenBank: AAA52509.1). The mature protein, predicted from this cDNA sequence in 458 amino acids, displays between 30 and 38% amino acid similarity to the previously identified GABA_A_ subunits [29]. Using the program Basic Local Alignment Search Tool (BLAST; https://blast.ncbi.nlm.nih.gov/Blast.cgi) [43], and the database of the Protein Data Bank, the structural model was built based on the structure of a human gamma-aminobutyric acid receptor, the Gaba(a) r-beta3 homopentamer (4COF.pdb) [44], which revealed a coverage of 70% and the highest sequence identity (48%) with the target. A multiple alignment was performed using the constraint-based multiple alignment tool (COBALT; https://www.ncbi.nlm.nih.gov/tools/cobalt/re_cobalt.cgi) server, where an initial segment of 44 amino acids with no structural identity appears. An ab initio model of this fragment was made using the Rosetta 3.4 program [45]; 1000 models of this segment were made. Subsequently, using MODELLER 9.17 r10881 with the multiple models protocol, 500 GABA_A_ models were constructed, using the most stable model of the peptide and the 4COF.pdb file as a template. Later, a simple structural refinement of full atom was performed using “*relax*” application of Rosetta. The final model was validated using the Verify-3D (structure evaluation software) and Whatcheck (protein verification tools software) computer programs [46, 47].

### Docking protocol

Docking was made with the previously constructed three-dimensional structure (Type A gamma-aminobutyric acid receptors—GABA_A_), and the ligands were built with HyperChem 8 software. The ligands were minimized employ Gaussian 09, revision A.02 (Gaussian Inc., Wallingford, CT) at DTF B3LYP/3-21G level of theory. AutoDockTools 1.5.4 (http://mgltools.scripps.edu/), it was used to prepare the structures before carrying out the docking. The preparation of the structures consisted in adding all hydrogen atoms as well as the Kollman united-atom partial charges to the receptor, while Gasteiger–Marsili charges and rotatable groups were assigned automatically to the ligands. AutoGrid4 was occupying to generate the electrostatic grid maps for each atom type. The initial grid box size was 60 Å × 60 Å × 60 Å in the x, y, and z dimensions. Docking was produced out with AutoDock4 version 4.2 (http://autodock.scripps.edu/) [48, 49] using default parameters; for the Lamarckian genetic algorithm with local search, number of individuals in population (150), maximum number of energy evaluations (2.5 million), maximum number of generations (27,000), rate of gene mutation (0.02), rate of crossover (0.8), and 1000 runs for docking. Finally, the docking was analyzed with AutoDockTools using cluster analysis and PyMOL software [50].

## Additional files

**Additional file 1: Figure S1.** Effect of Homo on Hypo-induced currents in oocytes heterologously expressing GABA_C_-ρ1R. (A) Representative traces of currents induced by 30 µM, 3 mM and 30 mM Hypo and co-applied with Homo at the indicated concentrations. (B) Homo dose-response relation of currents elicited by 30 µM, 3 mM and 30 mM Hypo. The currents were normalized to the maximum amplitude elicited by the agonist in absence of modulators. Data points are the means ± S.E. from at least 9 oocytes (n = 9) from 4 frogs (N = 4).

**Additional file 2: Figure S2.** Three-dimensional models of GABA_A_ and template. In slate blue cartoon (GABAA), splitpea green cartoon (template) (Protein Data Bank [PDB] code: 4COF). The structures were drawn using the PyMOL program.

## Abbreviations

GABA: γ-aminobutyric acid
Taurine: 2-Aminoethanesulfonic acid
Homotaurine: 3-Aminopropane-1-sulfonic acid
Hypotaurine: 2-Aminoethanesulfinic acid
Isethionic acid: 2-hydroxyethanesulfonic acid
TPMPA: Methyl(1,2,5,6-tetrahydropyridine-4-yl) phosphinic acid
MS-222: Ethyl 3-aminobenzoate methanesulfonate
NMDA: N-methyl-d-aspartate
HEPES: 4-(2-Hydroxyethyl)piperazine-1-ethanesulfonic acid
SCC-tau: sulfur-containing compounds structurally related to taurine
