# Supplementary material for: Differential modulation of human GABAC-ρ1 receptor by sulfur-containing compounds structurally related to taurine
Source: BMC Neurosci. 2018 Aug 3;19:47. doi: 10.1186/s12868-018-0448-6 (PMC6076408; doi:10.1186/s12868-018-0448-6)
Supplement: Supplementary file 1 — Additional file 1: Figure S1. Effect of Homo on Hypo-induced currents in oocytes heterologously expressing GABAC-ρ1R. (A) Representative traces of currents induced by 30 µM, 3 mM and 30 mM Hypo and co-applied with Homo at the indicated concentrations. (B) Homo dose-response relation of currents elicited by 30 µM, 3 mM and 30 mM Hypo. The currents were normalized to the maximum amplitude elicited by the agonist in absence of modulators. Data points are the means ± S.E. from at least 9 oocytes (n = 9) from 4 frogs (N = 4). [file 12868_2018_448_MOESM1_ESM.pptx]

## Slide 1
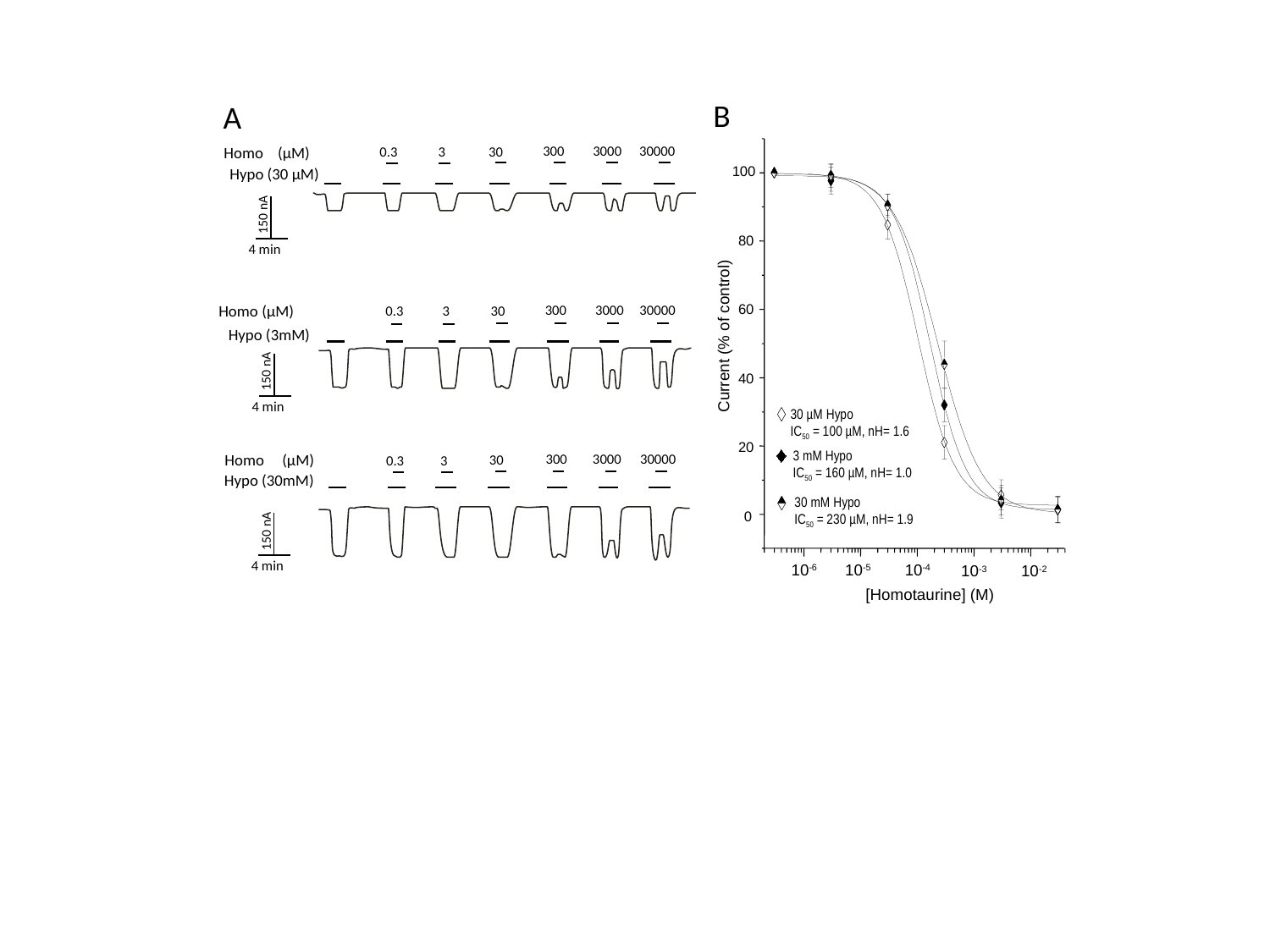

B
A
3000
30000
300
30
0.3
3
Homo (µM)
Hypo (30 µM)
150 nA
4 min
100
80
60
40
20
0
Current (% of control)
 30 µM Hypo
 IC50 = 100 µM, nH= 1.6
 3 mM Hypo
 IC50 = 160 µM, nH= 1.0
 30 mM Hypo
 IC50 = 230 µM, nH= 1.9
10-4
10-6
10-5
10-2
10-3
[Homotaurine] (M)
3000
30000
300
30
0.3
3
Homo (µM)
Hypo (3mM)
150 nA
4 min
Homo (µM)
Hypo (30mM)
3000
30000
300
30
0.3
3
150 nA
4 min
