# Supplementary figures and images for: Differential modulation of human GABAC-ρ1 receptor by sulfur-containing compounds structurally related to taurine
Source: BMC Neurosci. 2018 Aug 3;19:47. doi: 10.1186/s12868-018-0448-6 (PMC6076408; doi:10.1186/s12868-018-0448-6)

## Slide 1
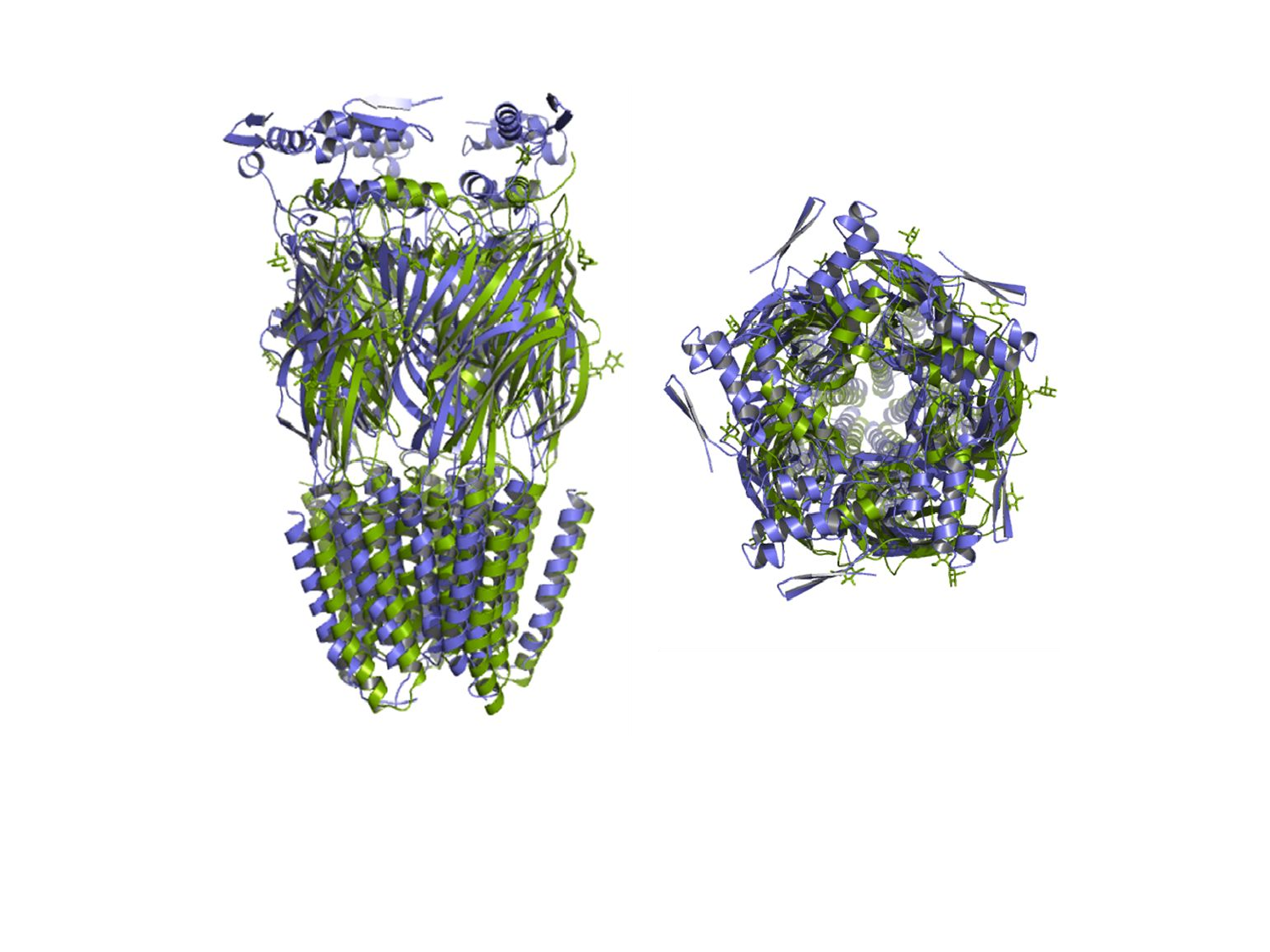

Supplement: Supplementary file 2 — Additional file 2: Figure S2. Three-dimensional models of GABAA and template. In slate blue cartoon (GABAA), splitpea green cartoon (template) (Protein Data Bank [PDB] code: 4COF). The structures were drawn using the PyMOL program. [file 12868_2018_448_MOESM2_ESM.pptx]
